# Treatment outcomes in patients with relapsed Graves’ disease

**DOI:** 10.20945/2359-4292-2026-0045

**Published:** 2026-04-01

**Authors:** Juliana de Andrade Carlini, Roberto Bernardo dos Santos, Nicolas Perini, João Hamilton Romaldini, Danilo Villagelin

**Affiliations:** 1 Curso de Pós-Graduação em Medicina Interna, Universidade Estadual de Campinas, Campinas, SP, Brasil; 2 Universidade São Francisco, Faculdade de Medicina, Bragança Paulista, SP, Brasil; 3 Endocrinologia e Metabologia, Hospital PUC-Campinas, Campinas, SP, Brasil; 4 Curso de Pós-Graduação em Ciências da Saúde, Hospital do Servidor Público Estadual, São Paulo, SP, Brasil; 5 Pontifícia Universidade Católica de Campinas (PUC-Campinas), Escola de Ciências da Vida, Faculdade de Medicina, Campinas, SP, Brasil

**Keywords:** Graves’ disease, hyperthyroidism, thyroid eye disease

## Abstract

**Objective:**

The objective of this study was to assess the outcomes of various treatment strategies in patients with relapsed Graves’ disease (GD) after standard antithyroid drug therapy (ATD). The three treatment strategies evaluated were radioiodine therapy (RAI), continuous low-dose methimazole (MMI), and a second course of MMI.

**Subjects and methods:**

This was a retrospective cohort study of 330 patients with GD who received initial MMI therapy for 12 to 24 months, of whom 159 subsequently relapsed. The cohort was categorized into three groups: 39 patients received RAI therapy plus levothyroxine, 46 patients received continuous low-dose MMI, and 74 patients received a second course of MMI. The analysis included thyroid function monitoring, progression of thyroid eye disease (TED), quality-of-life measures, and changes in body weight during follow-up. **Results:** The group receiving continuous low-dose MMI had a longer period of euthyroidism compared with the other groups. The TED outcomes and body weight changes were similar across all groups. Quality of life did not differ significantly across groups. Notably, 45% of patients in the group receiving a second MMI course relapsed when MMI was discontinued after a mean treatment duration of approximately 42 months.

**Conclusion:**

The administration of continuous low-dose MMI is an alternative for managing patients with relapsed GD, particularly for those who prefer to avoid definitive treatments.

## INTRODUCTION

Graves’ disease (GD) is the most prevalent cause of hyperthyroidism in iodine-sufficient regions, with an annual incidence of 20 to 50 cases per 100,000 ^(1)^. It can manifest at any age; however, the peak incidence typically occurs in patients aged 30 to 60 years ^(1)^, with a notably higher incidence among African Americans ^(2)^ and occurring more frequently in women ^(3)^.

Antithyroid drugs (ATDs) are the first-line treatment for GD worldwide ^(1,4-10)^; they inhibit thyroid hormone synthesis and may exert both direct and indirect effects on the immune system ^(10)^. The most frequent side effects are mild and include skin rash, arthralgia, and urticaria, occurring in 1%-5% of cases. Rare but severe side effects occur in less than 0.5% of cases and include agranulocytosis, fulminant hepatitis, and cholestatic jaundice ^(10,11)^; these are typically associated with high ATD doses and occur within the first 6 months of treatment ^(10)^.

According to the latest guidelines ^(12,13)^, the recommended duration of initial ATD therapy is 12-18 months, with a maximum remission rate of 50%-60% observed within this timeframe. Patients exhibiting persistently positive TSH receptor antibodies (TRAb) after 12-18 months may continue ATD therapy; otherwise, options include radioiodine (RAI) therapy or thyroidectomy. A meta-analysis assessing the use of ATD over a mean period of 5 years indicated that long-term treatment is safe, particularly at low doses in adults, suggesting it should be considered as an alternative for GD treatment; this approach is associated with minor complication rates, ranging from 2%-36%, while more serious complications are associated with higher doses, the use of propylthiouracil, and treatment in pediatric populations ^(14)^.

The long-term use of low ATD doses appears to be a viable option for patients who prefer to avoid RAI or thyroidectomy because of radiation exposure and potential postoperative complications, respectively ^(15,16)^. Some authors propose low ATD doses in GD as an alternative treatment for selected groups, analogous to other chronic diseases ^(17)^.

The primary objective of this study was to evaluate the effectiveness of the following treatment modalities in patients with GD who received standard ATD therapy for 12-24 months: ^(1)^ continuous use of a low MMI dose (MMIc), ^(2)^ a second course of ATD therapy (MMIs) after relapse, continued until TRAb became negative, and ^(3)^ RAI plus levothyroxine replacement. Notably, none of the patients in this study underwent surgery for GD treatment. Secondary study objectives include evaluating the safety of the three treatments, comparing the three groups in terms of thyroid eye disease (TED) outcomes, and assessing the patients’ quality of life and changes in body weight. Predictors of remission were also analyzed in the MMIs group.

## SUBJECTS AND METHODS

### Patients with Graves’ disease

This was a retrospective study conducted at a Brazilian university hospital center in accordance with the ethical principles of Good Clinical Practice, the Declaration of Helsinki, and the International Council for Harmonisation (ICH) E6 guidelines. The study was approved by the institution’s Ethics Committee under the approval number CAAE 39062520.8.0000.5481. The study complied with the countries’ legal requirements and received approval from an institutional review board, with all patients providing signed informed consent. In total, 330 patients diagnosed with GD who underwent treatment with methimazole (MMI) for 12-24 months were evaluated.

The diagnosis of GD was based on the presence of a diffuse goiter on physical examination and confirmed by thyroid ultrasound, together with decreased TSH and elevated free T4 levels. The etiology of hyperthyroidism was established through positive TRAb levels or diffuse iodine uptake on thyroid scintigraphy. Relapse was defined as the reappearance of clinical symptoms of hyperthyroidism after discontinuation of MMI, confirmed by laboratory tests (TSH suppression and elevated free T4), after the end of treatment, with positive TRAb. Remission was defined as persistent clinical and laboratory euthyroidism for more than 1 year after MMI interruption. Exclusion criteria included age below 18 years, use of propylthiouracil for hyperthyroidism management, pregnancy or lactation, and thyroidectomy or RAI as primary treatment.

### Study design

Data on 330 consecutive patients with GD were retrospectively reviewed. The patients were treated with MMI for 12-24 months, and the drug was discontinued in those who tested negative for TRAb. A total of 159 patients experienced relapse after ATD interruption. These patients were offered three treatment options: ^(1)^ RAI therapy (RAI group) plus levothyroxine replacement, ^(2)^ reintroduction of MMI in continuous low dose uninterruptedly, using the lowest MMI dose to maintain euthyroidism during follow-up (MMIc group), and ^(3)^ a second MMI course or at least 24 months or until TRAb became negative (MMIs group). Using a standardized algorithm, D.V. or R.B.S. (both authors and physicians) discussed the advantages and disadvantages of each treatment with the patients, allowing the patients to make the final decision.

In the RAI group (39 patients), individuals were informed of the potential side effects of RAI. A fixed RAI dose of 15 mCi was used, and levothyroxine replacement was initiated when TSH levels exceeded 5.0 mIU/L. All patients developed hypothyroidism after 6 months. The MMIc group (46 patients) received MMI in continuous low doses to maintain euthyroidism. The MMIs group (74 patients) received a second MMI course for at least 24 months or until testing negative for TRAb. Patients in both MMI groups were informed of the potential side effects of MMI and symptoms of thyrotoxicosis.

All patients underwent clinical and laboratory evaluations during the first year, every 3 months, and subsequently every 6 months during routine consultations. Data collected included smoking status, symptoms of hyperthyroidism, and other comorbidities (diabetes, hypertension, arrhythmia, cardiovascular events, and other autoimmune diseases). Body weight, TED, and side effects associated with the medication were assessed for those receiving treatment. Laboratory tests, including TSH, free T4, white blood cell count, serum aspartate aminotransferase, serum alanine aminotransferase, gamma-glutamyl transferase, fasting glucose, and creatinine, were conducted at each visit. All patients were encouraged to adhere to and maintain their treatments throughout the follow-up period.

### Weight evaluation

Patient weight was measured in routine medical appointments using certified scales. Prior to weighing, patients were instructed to remove shoes, coats, and light jackets.

### Thyroid eye disease evaluation

Assessment of TED was performed using the Clinical Activity Score (CAS) as described by Morrits and cols. ^(18)^. This scoring system evaluates seven TED manifestations: eyelid edema, conjunctival edema (chemosis), caruncle edema, conjunctival hyperemia, eyelid erythema, spontaneous retrobulbar pain, and pain due to eye movement. One point is assigned for each manifestation, ranging from zero (no activity) to seven (very high activity). Palpebral fissure, visual acuity, and intraocular pressure were measured. Proptosis was assessed using a Hertel exophthalmometer, and diplopia was classified as constant, inconstant, and intermittent. Worsening of CAS was defined as an increase of at least 2 points from baseline, and inflammation was considered active when CAS was > 3. Patients in the RAI group with CAS > 3 were treated with prednisone at a dose of 0.5 mg/kg/day, followed by tapering over 30-40 days until discontinuation ^(19)^.

### Quality-of-life assessment

Quality of life was assessed at the end of the study using ThyPRO-39 ^(20)^, a questionnaire specific to thyroid diseases. This questionnaire consists of 39 items that evaluate signs and symptoms of thyroid disease across multiple domains, including hyperthyroidism, hypothyroidism, goiter, TED, fatigue, cognitive decline, anxiety, depression, social impairment, daily and sexual life, and aesthetic concerns. Each item is rated on a 5-point Likert scale (0 = not at all; 1 = a little; 2 = moderately; 3 = quite a bit; 4 = very much). Domain and overall scores are then linearly transformed to a 0-100 scale, with higher scores indicating greater symptom burden and poorer quality of life. The questionnaire was administered exclusively to patients with euthyroidism.

### Thyroid dysfunction and TRAb evaluations

Thyroid function was evaluated during routine medical appointments by measuring TSH and free T4 levels, which were monitored throughout follow-up. Thyroid function status was categorized as euthyroidism (TSH and free T4 levels within reference values), overt hyperthyroidism (TSH levels below and free T4 elevated above reference values), subclinical hyperthyroidism (TSH levels below and free T4 within reference values), overt hypothyroidism (elevated TSH levels and free T4 below reference values), and subclinical hypothyroidism (elevated TSH levels with free T4 within reference values). Thyroid function tests were performed every 3 months during the first year, then every 6 months thereafter. The percentage of periods in euthyroidism was calculated by dividing the number of times thyroid function was within the euthyroid range by the total number of thyroid function assessments performed during the study period. In the MMIs and MMIc groups, TRAb levels were measured yearly until they became negative.

### Laboratory analysis

Reference values for serum measurements included TSH (0.4-4.5 mIU/L) and free T4 (0.8-1.9 ng/dL or 10.3-24.5 pmol/L), as measured using the Elecsys system (Roche Diagnostics, Rotkreuz, Switzerland). Serum TRAb levels were considered positive when > 10 IU/mL, as assessed using a radioreceptor assay (RSR Limited, Cardiff, United Kingdom). Thyroid size was evaluated by ultrasound (GE Healthcare, Chicago, IL, USA) and was considered normal when measuring 6-15 mL. Thyroid 99mTc-pertechnetate uptake (Siemens Healthineers, Erlangen, Germany) was considered normal within the reference range of 0.35%-1.7%. Thyroid peroxidase antibodies (TPOAb; positive when > 35 IU/mL) and antithyroglobulin antibodies (TgAb; positive when > 40 IU/mL) were measured routinely at baseline using a chemiluminescence assay (Elecsys system, Roche Diagnostics). Plasma glucose levels were measured using the oxidase method, and serum creatinine and liver enzymes were measured using colorimetric and enzymatic assays, respectively (Roche Diagnostics).

### Statistical analysis

A descriptive analysis presents frequency tables for categorical variables and measures of central tendency and dispersion for numerical variables. The Mann-Whitney test was applied for comparisons between two groups, whereas the Kruskal-Wallis test was used for comparisons among three groups, followed by Dunn’s *post hoc* test for multiple comparisons when appropriate. The chi-square test or Fisher’s exact test was employed as required. Analysis of variance (ANOVA) was performed to compare weight change across age groups. A significance level of 5% was adopted for all statistical tests. Analyses were performed using SAS software, version 9.4 (SAS Institute Inc., Cary, North Carolina, USA).

## RESULTS

**Figure 1:**
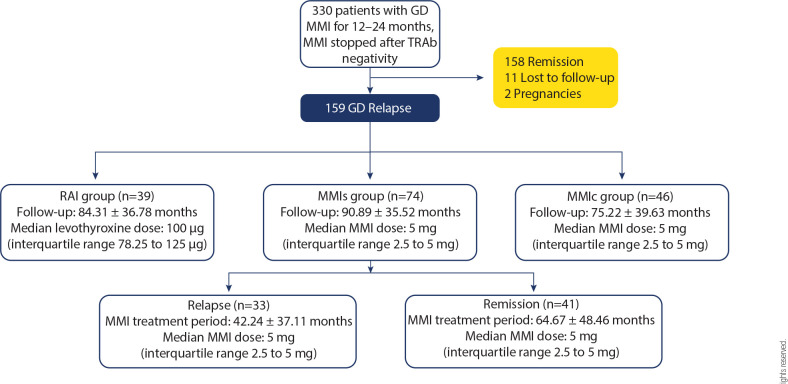
Study design and distribution of patients with Graves’ disease according to treatment.

The study design and patient distribution by treatment are shown in **Figure 1**.

Mean values are presented with standard deviation, and median values are presented with interquartile range. GD: Graves’ disease; RAI: radioiodine therapy; MMIc: continuous low-dose methimazole; MMIs: second course of methimazole after relapse continued until TRAb became negative.

**Table 1** describes the baseline characteristics of the patients at relapse, after an initial course of ATD. No significant differences among groups were observed regarding age, sex, smoking status, thyroid function, positive thyroid antibodies, TED evaluated by the CAS scoring system, and body weight. Measurement of TRAb, obtained when patients relapsed after the initial ATD course, showed positive results in 97.4% of patients in the RAI group, 95.7% in the MMIc group, and 100% in the MMIs group (p = NS). Three patients with negative TRAb had relapsed disease, one in the RAI group and two in the MMIc group.

**Table 1:** Initial baseline characteristics of the three groups

Parameters	RAI group	MMIc group	MMIs group	P value
Number, n	39	46	74	
Age, years	45.10 ± 14.00	46.02 ± 13.34	46.04 ± 13.16	
Sex (F/M ratio), n	34/5	35/11	65/9	NS
Smokers, %	33%	28.3%	31.1%	NS
TSH level, mIU/L	0.024 ± 0.084	0.021 ± 0.046	0.015 ± 0.031	NS
Free T4, ng/dL	3.87 ± 1.87	3.88 ± 2.26	3.96 ± 2.37	NS
Positive TRAb, %	97.4%	95.7%	100%	NS
Positive TPOAb, %	68.8%	58.5%	70.6%	NS
Positive TgAb, %	46.7%	40%	41.2%	NS
Thyroid US, mL	37.40 ± 48.13	20.17 ± 12.39	21.55 ± 12.56	NS
CAS	0.62 ± 1.04	0.76 ± 1.21	0.47 ± 1.06	NS
CAS 0, n	27	30	56	
CAS 1, n	4	5	10	
CAS 2, n	6	5	5	
CAS 3, n	1	4	0	
CAS 4, n	1	2	1	
CAS 5, n	0	0	2	
CAS 6, n	0	0	0	
CAS 7, n	0	0	0	
Weight, kg	59.08 ± 14.68	67.20 ± 13.55	65.82 ± 12.40	NS
Follow-up, months	84.31 ± 36.78	75.2 ± 39.6	90.89 ± 35.52	

Data are shown as mean ± standard deviation or percentage (%).F: female; M: male; RAI: radioiodine therapy; MMIc: continuous low-dose methimazole; MMIs: second course of methimazole after relapse until TRAb became negative; TSH: thyrotropin; FT4: free thyroxine; TRAb: thyroid receptor antibody; TPOAb: thyroperoxidase antibody; TgAb: thyroglobulin antibody; US: ultrasound measurement of thyroid volume; CAS: clinical active score; kg: kilogram; NS: not significant.

The mean follow-up durations in the MMIc, MMIs, and RAI groups were 75.2 ± 39.6 months, 90.9 ± 35.5 months, and 84.3 ± 36.8 months, respectively (p = NS). The median MMI dose in the MMIc and MMIs groups was 5 mg/day (interquartile range [IQR] 2.5-5 mg), and the median levothyroxine replacement dose in the RAI group was 100 µg/day (IQR 78.25-125 µg) (**Figure 1**).

Thyroid function was evaluated in the three groups every 3 months during the first year and every 6 months thereafter. The percentage of periods in euthyroidism was calculated by dividing the number of times thyroid function was within the euthyroid range by the total number of thyroid function assessments performed during the study period in each group. Euthyroidism was more frequent in the MMIc group (78.7%) compared with the RAI group (51.2%, which notably received levothyroxine replacement), and the MMIs group (55.4%) (p < 0.05). No side effects were observed with prolonged MMI use or with RAI treatment.

**Figure 2:**
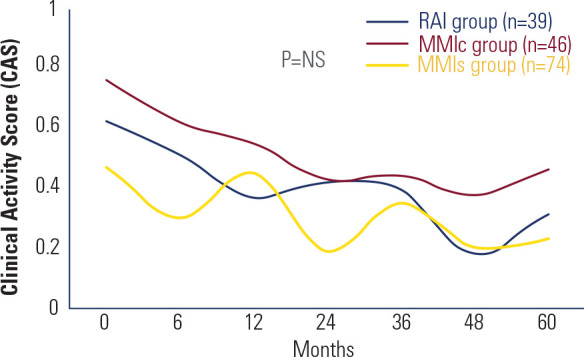
Progression of the Clinical Activity Score (CAS) during follow-up.

RAI: radioiodine therapy; MMIc: continuous low-dose methimazole; MMIs: second course of methimazole after relapse continued until TRAb became negative; NS: not significant.

**Figure 3:**
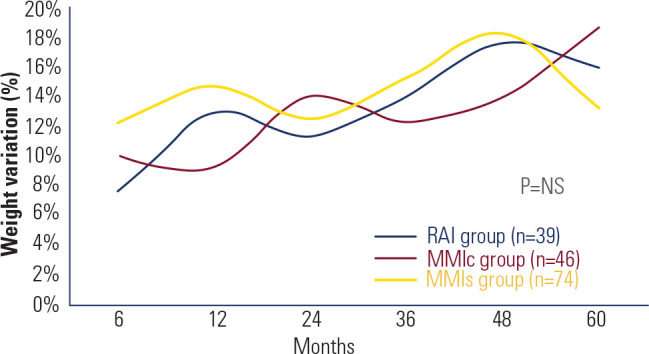
Percentage of weight variation during follow-up in the three groups.

RAI: radioiodine therapy; MMIc: continuous low-dose methimazole; MMIs: second course of methimazole after relapse continued until TRAb became negative; NS: not significant.

No significant differences in mean CAS values were observed among the three groups at baseline or during follow-up (**Figure 2**). Quality of life - evaluated in euthyroid patients at the end of the study using the ThyPRO-39 questionnaire - was similar across all three groups. **Figure 3** illustrates the percentage weight variation across the three groups during the study. All groups experienced weight gain during treatment of hyperthyroidism, but no significant differences were observed among them during follow-up. Additionally, weight gain was evaluated in relation to participants’ age to assess its influence. The ANOVA test showed no statistically significant differences among the three groups across all periods.

Regarding cardiovascular disease, there were no significant differences between the groups. Acute coronary syndrome was present in two patients in the MMs and RAI group and in one patient in the MMI group. Atrial fibrillation occurred in one case in the RAI and MMIc groups. The three groups had two patients with ischemic stroke. No side effects related to MMI use were observed in the MMIc and MMIs groups.

### Remission rates in the MMIs group

All patients in the MMIs group were euthyroid and tested negative for TRAb when ATD was discontinued after the second treatment course at a mean treatment duration of 54.39 ± 44.77 months. Among the 74 patients in this group, 33 (44.6%) relapsed at a mean of 12.36 ± 10.87 months after MMI interruption. **Table 2** outlines the characteristics of these patients at cessation of the second MMI course; no significant differences were observed between the remission and relapse groups with respect to sex, age, smoking status, thyroid antibodies, or TED evaluated by CAS. However, treatment duration in the remission group (64.67 ± 48.46 months) was longer than in the relapse group (42.24 ± 37.11 months; p < 0.01). The mean follow-up duration after MMI interruption in the remission group was 36.5 ± 6.29 months. Notably, quality-of-life was evaluated in the MMIs group in patients who were euthyroid, those with remission, and those who relapsed and resumed MMI therapy.

**Table 2:** Clinical characteristics of patients who experienced relapse versus remission upon cessation of methimazole (MMI)

Parameters	Relapse	Remission	P value
Number, n	33	41	
Age, years	44.97 ± 14.71	46.90 ± 11.88	NS
Sex (F/M ratio), n	30/3	35/6	NS
Smokers, %	24.2%	36.6%	NS
Positive TRAb, %	100%	100%	NS
Positive TPOAb, %	62.1%	76.9%	NS
Positive TgAb, %	42.9%	40%	NS
Thyroid US, mL	21.76 ± 12.37	21.39 ± 12.88	NS
CAS	0.30 ± 0.77	0.60 ± 1.24	NS
CAS 0, n	26	29	
CAS 1, n	6	4	
CAS 2, n	0	5	
CAS 3, n	0	0	
CAS 4, n	1	0	
CAS 5, n	0	2	
CAS 6, n	0	0	
CAS 7, n	0	0	
MMI use, months	42.24 ± 37.11	64.67 ± 48.46	0.0065
Weight, kg	67.51 ± 14.15	64.74 ± 11.31	NS
Follow-up, months	93.38 ± 38.64	88.95 ± 33.24	

Data are shown as mean ± standard deviation or percentage (%).F: female; M: male; TRAb: thyroid receptor antibody; TPOAb: thyroperoxidase antibody; TgAb: thyroglobulin antibody; US: ultrasound measurement of thyroid volume; CAS: clinical active score; kg: kilogram; NS: not significant.

## DISCUSSION

The management of GD has long posed a clinical challenge. Recent guidelines from both the American Thyroid Association ^(13)^ and the European Thyroid Association ^(12)^ support the consideration of a second course or prolonged ATD use in patients who experience disease relapse. Moreover, a recent international survey indicated that over 90% of respondents favored ATD therapy as the initial treatment choice for a typical case of uncomplicated GD ^(21)^. This survey ^(21)^ further investigated treatment preferences in cases of GD relapse, revealing that 60% of clinicians selected ATDs as the first-line approach, followed by RAI at 37%, and surgery at just 3%. Nevertheless, the evidence base for these preferences remains limited. Few studies have directly compared ATD and RAI therapies in relapsed GD patients, and the optimal duration of a subsequent ATD course, as well as its impact on long-term remission rates, has yet to be established. The present study aimed to address this clinically important and underexplored issue.

A real-world study conducted in Sweden, with a long-term follow-up of ATD treatment for GD, reported relapse rates of 59.7% over a follow-up period of 6-10 years ^(22)^. These findings are comparable to those of the present study, in which 159 out of an initial cohort of 330 patients experienced relapses, corresponding to a 48% relapse rate. Notably, similar relapse rates were observed in patients who used MMI in the first and second cycles. The MMIs group, after a mean duration of MMI use of approximately 6 years, had a 45% relapse rate.

In contrast, Azizi and cols. ^(23)^ reported a significantly lower relapse rate in a group of patients treated with long-term ATD (8 years; relapse rate 15%) compared with another group treated with short-term ATD (approximately 2 years; relapse rate 53%) during a mean follow-up of 4 years. A relationship between ATD therapy duration and remission rates has also been reported in a Korean study ^(24)^, which found remission rates of 57% for 12 months of ATD treatment and 81% for 72 months.

The discrepancies between these findings may stem from differences in genetic predisposition or environmental factors among the study populations. Additionally, the patients in the present study experienced relapse after initial treatment, whereas those in the study by Azizi and cols. ^(23)^ and the Korean study received continuous ATD therapy for over 5 years. It is possible that ongoing ATD treatment, while maintaining euthyroidism, positively influences the autoimmune system.

The ultimate goal of GD treatment is to control thyroid function, as thyroid dysfunction is associated with deleterious effects on quality of life, morbidity, and mortality, even in subclinical forms ^(25-27)^. The current study demonstrated that euthyroidism was more frequent in the MMIc group (78%) than in the RAI group (plus levothyroxine replacement; 51%) and the MMIs group (55%) over a follow-up period exceeding 6 years. Several studies have evaluated thyroid function control in patients with hypothyroidism from various etiologies, reporting euthyroidism rates of 60%-65% ^(28-30)^, highlighting the challenges in managing patients with hypothyroidism. Other cohorts describe lower euthyroidism rates with the use of RAI and levothyroxine replacement when compared with ATD ^(5)^. Why thyroid function is better controlled with MMI (continuous, low-dose) than with levothyroxine remains an intriguing question.

The results in the MMIs group can be viewed as a “glass-half-full/glass-half-empty” dilemma. On the one hand, nearly half of the patients achieved remission and no longer required ATD; on the other hand, a substantial proportion relapsed after stopping ATD, exposing them to the adverse effects of hyperthyroidism, necessitating the reintroduction of ATD. Remission rates in this group were 55%, and longer treatment duration was identified as the sole variable distinguishing patients who relapsed from those who remained in remission. Notably, TRAb was negative in both groups when MMI was discontinued and was not a reliable prognostic marker. Although no direct linear relationship exists, the data suggest that increased treatment duration correlates with higher remission rates ^(31)^. Wiersinga and cols. recently proposed that long-term treatment with ATDs (*i.e*., 5-10 years) is feasible and associated with fewer recurrences (15%) compared with short-term treatment (*i.e*., 12-18 months) ^(32)^.

Analysis of TED across the three groups revealed no differences in progression or resolution during the follow-up period. Several key aspects warrant emphasis: most patients had either no TED or only mild TED; patients in the RAI group with risk factors for deteriorating TED post-RAI received glucocorticoid prophylaxis in accordance with established guidelines ^(33)^. The results of the present study reinforce the safety and potential benefits of long-term ATD treatment for TED.

Quality of life and body weight gain are two critical concerns for patients with thyroid dysfunction. Analysis using the ThyPRO-39 questionnaire indicated no significant difference in quality of life among the groups. To avoid confounding variables, data were analyzed exclusively in patients with euthyroidism. Furthermore, body weight increased in all three groups, with no significant differences among them. Previous studies have investigated body weight changes associated with GD treatments ^(5,34)^. Treatment with RAI appears to be associated with significantly greater weight gain than ATD, likely influenced by thyroid function control ^(35)^. Ultimately, the present study reinforces the safety of prolonged MMI use, consistent with findings from recent meta-analyses ^(15)^.

This study has some limitations. As this was a retrospective study, it may be subject to bias, and the findings should be interpreted with caution. Additionally, the quality-of-life questionnaire was administered only at the end of the study, rather than at baseline, which affected data interpretation. Furthermore, due to the small number of patients with moderate-to-severe TED, we were unable to evaluate how patients with more severe forms of TED would respond to the three treatment options.

This study aimed to address an essential question in clinical practice: Do patients who relapse from GD and restart ATD require a second course of ATD (to aim for remission) or should they remain on a continuous low-dose of ATD? To our knowledge, this is the first study comparing a second course of MMI with continuous MMI and RAI treatments in patients with relapsed GD. This represents a real-world dilemma for clinicians and patients when deciding on subsequent treatment strategies following GD relapse.

In conclusion, our study demonstrated that continuous long-term use of low-dose MMI may be an alternative treatment option for patients who relapse after initial ATD treatment for 12-18 months, and is associated with improved thyroid function control and no differences in TED outcomes, quality of life, or body weight changes compared with a second course of MMI or RAI therapy. Additionally, if a second course of ATD is chosen after ATD is interrupted and TRAb is negative, it is recommended to extend treatment duration to longer periods (> 5 years), as a shorter second MMI course may be associated with relapse rates similar to those of the initial course.

## Data Availability

datasets related to this article will be available upon request to the corresponding author.
